# Astrochemically Relevant Radicals and Radical–Molecule Complexes: A New Insight from Matrix Isolation

**DOI:** 10.3390/ijms241914510

**Published:** 2023-09-25

**Authors:** Vladimir I. Feldman

**Affiliations:** Department of Chemistry, Lomonosov Moscow State University, Moscow 119991, Russia; feldman@rad.chem.msu.ru

**Keywords:** matrix isolation, free radicals, radical–molecule complexes, astrochemistry, radiation chemistry, infrared spectroscopy, electron paramagnetic resonance, cryogenic tempeartures

## Abstract

The reactive open-shell species play a very important role in the radiation-induced molecular evolution occurring in the cold areas of space and presumably leading to the formation of biologically relevant molecules. This review presents an insight into the mechanism of such processes coming from matrix isolation studies with a main focus on the experimental and theoretical studies performed in the author’s laboratory during the past decade. The radicals and radical cations produced from astrochemically relevant molecules were characterized by Fourier transform infrared (FTIR) and electron paramagnetic resonance (EPR) spectroscopy. Small organic radicals containing C, O, and N atoms are considered in view of their possible role in the formation of complex organic molecules (COMs) in space, and a comparison with earlier results is given. In addition, the radical–molecule complexes generated from isolated intermolecular complexes in matrices are discussed in connection with their model significance as the building blocks for COMs formed under the conditions of extremely restricted molecular mobility at cryogenic temperatures.

## 1. Introduction

The problem of cold chemical evolution leading to the formation of complex organic molecules (COMs) and prebiotic species in various objects of our Universe, including interstellar media (ISM) and cometary and planetary ices, is one of the key issues of molecular astrophysics, astrochemistry, and astrobiology [1]. Generally speaking, it is obvious that high-energy cosmic radiation plays a very important role in primary chemical reactions occurring in icy space media [2,3], and many of these processes involve the intermediate formation of free radicals and radical ions. The impact of radical chemistry on the processing of astrophysical ices was discussed in a large number of experimental works (see, e.g., Refs. [4,5,6,7,8,9] for recent examples). Nevertheless, direct observations of primary highly reactive radicals in the laboratory ice simulation experiments are limited, and the dynamics and reactivity of these species under conditions of extremely restricted molecular mobility are poorly understood. Matrix isolation is a useful complementary approach to obtaining information on detailed and precise spectroscopic characteristics of astrochemically important radicals, suitable for comparison with astronomical observational data. Actually, the spectroscopic benefit for space science from matrix isolation was realized long ago [10]. An interesting illustrative example of astrochemically relevant studies of radicals using matrix isolation with combined spectroscopic detection can be found in a recent work [11]. The overall progress in this field is outlined in a recent review [12], which demonstrated that matrix isolation can also provide rich (and sometimes unique) information on the reaction mechanisms in synthetic astrochemistry induced by high-energy radiation in cosmic ices. The present review is mainly focused on the experimental and computational studies on astrochemically relevant small organic radicals (both neutral and charged) and radical–molecule complexes generated by ionizing radiation in solid noble gas matrices, which were performed in our laboratory during recent years. The basic goal is to highlight new implications of matrix isolation for a better understanding of the role of radical species in key processes in astrochemistry (and beyond it) and to discuss related problems and new prospects.

## 2. Approaches and Methods

In order to realize the full potential of the matrix isolation technique for the studies of the radiation-induced radicals of astrochemical interest, we have to briefly consider common features of the physical and chemical processes occurring under irradiation of matrix-isolated species. They can be represented by the following general scheme [13,14,15]:

Ng → Ng^+**•**^, e^−^, Ng* (1)

Ng^+∙^ + RH → (RH^+**•**^)* + Ng (2)

Ng* + RH → RH* + Ng (3)

(RH^+**•**^)* → R_1_^+^ + R_2_**^•^** (4)

(RH^+**•**^)* → R’H^+**•**^ + M (5)

(RH^+**•**^)* → RH^+**•**^ (6)

RH^+**•**^ + e^−^ → RH** (7)

RH* (RH**) → R**^•^** + H**^•^** (8)

RH* (RH**) → non-radical products (9)

RH* (RH**) → RH (10)

Here, Ng is a matrix (noble gas) atom, and RH is a guest molecule, a potential precursor of COMs, which contains at least one hydrogen atom. Under the conditions of matrix isolation (Ng/RH~1000), the ionizing radiation (high-energy photons, electrons, or ions) is primarily absorbed by the matrix, resulting in a population of ionized and excited states of matrix atoms (there is a basic difference to note from selective resonance absorption of light by guest molecules in the case of UV or VUV photolysis). Processes (2) and (3) represent positive hole and excitation transfer leading to activation of the guest molecule. An important feature is that the hole transfer is usually highly exothermic because the difference between ionization energies (IE) of Ng and RH is typically from 1 to 7 eV for Xe, Kr, and Ar matrices (and even more in the case of Ne matrix). This IE gap results in the initial formation of electronically and vibrationally excited radical cations denoted as (RH^+**•**^)*, which may undergo either “hot” reactions to yield both radical and non-radical products denoted as processes (4) and (5) or relaxation to the ground-state radical cations (6). If the electrons are not captured by guest molecules (which is a rather specific case), they finally recombine with the radical cations, as represented by Reaction (7), resulting in the formation of secondary (recombination) electronic excited states denoted as RH**. The difference between RH* and RH** is concerned with two reasons: first, recombination may result in a population of high excited states (close to the ionization threshold), and second, it gives a high population of triplet excited states unattainable by direct excitation. The latter effect results from the loss of spin correlation in the recombining pairs (RH^+**•**^ + e^−^) because, in the matrix isolation experiment, positive holes and electrons are typically produced from different precursors separated by large distances. In such a situation, the triplet-to-singlet (T/S) ratio may approach the statistical value of 3/1. In any case, both RH* and RH** may undergo dissociation to radical and hydrogen atoms, give molecular (diamagnetic) products, or relax to the ground state, as depicted by processes (8), (9), and (10), respectively. In the above-considered scheme, we have ignored an opportunity for dissociation of the neutral excited state to two “heavy” radical fragments because such processes are usually suppressed in rigid cryogenic matrices due to the cage effect (with a few exceptions involving small radicals, which may get an excess kinetic energy sufficient to escape from the matrix cage). A specific case is represented by a *para*-hydrogen matrix, where radical fragments may escape the matrix cage, even at very low temperatures [16].

To sum up, the radicals can be produced from both ionized and excited states, the latter process most commonly giving hydrogen atoms and radicals retaining the initial molecular structure of the parent guest molecules (certainly, rearrangement may occur at the second stage). It is important to note that the matrix effect on the efficiency and mode of radical formation in the row of noble gas matrices may be very significant, and it originates from at least two factors. First, the efficiency of “hot” ionic fragmentation (4) increases with increasing the IE gap, that is, from xenon to argon and neon. Second, the efficiency of relaxation of both ionic and neutral excited states increases with increasing the matrix polarizability, that is, from neon to xenon. It should be taken into account not only for the interpretation of the results but also for the selection of a matrix to study specific effects (some examples will be shown below).

Generally speaking, the above-given scheme is valid for the generation of radicals in noble gas matrices using different kinds of ionizing radiation, including electrons and photons in a wide range of energy (from 10^3^ to 10^7^ eV). As mentioned above, it is different from the situation for radical production by UV or VUV photolysis in matrices, where direct absorption of photons by target molecules is a dominating process. It should be stressed that, in all the cases using high-energy ionizing radiation, the overwhelming majority of primary events are caused by secondary electrons of relatively low energy, so from the chemical point of view, there is no principal difference (for example, it was demonstrated that the product composition is similar for irradiation with 1 MeV electrons and X-rays with effective energy of ca. 20 keV [13,14]). This similarity justifies the application of convenient laboratory X-ray sources for qualitative modeling of the astrochemical processes, which are naturally induced by a wide range of space radiation. Certainly, one should bear in mind that the dose rate and dose-depth distribution may differ significantly for different kinds of radiation, so simulation of macroscopic kinetics and diffusion relevant to ice astrochemistry represents a special problem, which is out of the scope of this review.

Regarding the registration methods, the common practice of radical characterization in different systems implies using electron paramagnetic resonance (EPR) spectroscopy as a highly informative and selectively spin-sensitive technique. On the other hand, in matrix isolation studies, the most widely used method is definitely Fourier transform infrared (FTIR) spectroscopy, which is suitable for the detection of different kinds of species independent of their magnetic processes. It should be noted that, in certain cases, the application of FTIR spectroscopy makes it possible to observe the radicals, which are not unequivocally identified by EPR. This is particularly true for the radicals with strongly anisotropic g and hyperfine coupling tensors, which may give complex and broadened EPR signals in macroscopically disordered matrices (some examples will be discussed in the next section). Thus, the best way is to combine EPR and FTIR spectroscopic methods to get full information regarding the structure, dynamics, and reactions of the radiation-induced radicals in noble gas matrices [17]. Despite the fact that this powerful combination of complementary spectroscopic methods was effectively used in a number of matrix isolation studies (see, e.g., Refs. [11,18,19,20,21,22]), the examples of its application to the radicals produced from astrochemically relevant molecules are rather limited. This review will outline our recent efforts in this direction, focusing on comparison, limitations, and perspective. From an experimental point of view, the approach applied in our laboratory at Lomonosov Moscow State University (MSU) is based on using a complex of original cryostats described in detail elsewhere [17], which makes it possible to study the radiation-induced transformations of small matrix isolated molecules under the action of X-rays, VUV, and UV light (a previous version of continuous-flow cryostat used at Karpov Institute of Physical Chemistry (KIPC, 1995–2006) was designed for irradiation with fast electrons (~1 MeV). Some results of those studies are also covered in this review). The studies were extensively supported by high-level quantum chemical calculations, also being an important part of our strategy.

## 3. Radicals and Radical Ions Produced from Astrochemically Important Molecules

A number of small astrochemically relevant radicals were characterized by IR, UV, and EPR spectroscopy under the conditions of matrix isolation for several decades. It is worth noting that, in many cases, the corresponding works did not reflect specific astrochemical motivation, but some results of these studies have been successfully used for the identification of radicals observed in space (see Ref. [12] and references therein). In early works, the matrix-isolated radicals were generated by different methods, and the mechanism of their formation was typically not considered in detail. Here, we will mainly consider the formation of radicals from isolated molecules in deposited solid noble gas matrices under the action of high-energy radiation. Actually, this method is related to the basic modeling of the “cold” radiation-induced astrochemistry, which occurs under the procession of cosmic ices.

### 3.1. Hydrocarbon Radicals

The hydrocarbon radicals apparently play an important role in both prebiotic astrochemistry and the formation of polycyclic aromatic hydrocarbons (PAHs). It is worth noting that the simplest species of this kind, methylydine radical (CH^•^), was probably the first molecule reliably detected in space [23]. This fundamentally important diatomic species mostly belongs to the world of “hot” astrochemistry occurring in the star formation regions.

#### 3.1.1. Methyl Radical

The astronomical observation of methyl radical (CH_3_**^•^**) in the ISM was reported in 2000 [24] and, highly likely, it plays a noticeable role in ice astrochemistry, being a common product of the radiation-induced degradation of various organic molecules and a constituent for building new COMs. The structure and dynamics of this radical have been extensively studied in matrices by EPR spectroscopy (see, e.g., [25,26,27,28]), and its vibrational and electronic features were characterized by matrix isolation IR spectroscopy (the first observation was reported by Milligan and Jacox in 1967 [29], the compilation of data obtained in different matrices is available [30]). The manifestations of methyl radicals in the EPR spectra in matrices are very prominent, even at relatively low abundance, because it typically gives a characteristic quartet signal consisting of sharp lines (to note, the internal dynamics of this radical at very low temperatures have a strong effect on the intensity ratio in the quartet signal and results in specific splitting, which was a topic of experimental and theoretical studies [27,28,31,32,33]). Due to the extremely high sensitivity of the EPR detection of methyl radical, the observation of weak signals from this species in irradiated matrices is somewhat ambiguous because it can actually originate from unidentified impurities [34,35]. The most intense feature of methyl radical in the IR spectra corresponds to the out-of-plane (OPLA) deformation mode and appears in the region of 600–625 cm^−1^ (depending on the matrix [30]), other IR absorption bands of this radical produced by irradiation are weaker and may overlap with the bands of parent compounds.

In early studies, methyl radicals in the solid noble gas matrices were produced from methane by radiolysis or VUV photolysis [25,27,29]. Meanwhile, as shown by EPR spectroscopy, this species may also arise from larger saturated hydrocarbons [17,25]. Our studies using both EPR and IR spectroscopy showed that methyl radicals are produced under irradiation of a number of astrochemically relevant matrix-isolated molecules containing methyl group, such as methanol [36], ethanol [37], acetaldehyde [38,39], acetone [38], and acetonitrile [40]. It is worth noting that the methyl radical (detected by EPR) was also found to be produced by VUV photolysis of methanol in an argon matrix [11]. Obviously, these radicals result from the skeleton fragmentation of the parent molecules. Considering the radicals produced by high-energy radiation, two important points should be highlighted in this respect. First, within the row Ar-Kr-Xe, methyl radicals are most effectively produced in argon, while their yield in xenon is small or even negligible, and krypton represents an intermediate case (limited data are available for a neon matrix). Second, molecular structure plays an important role in the fragmentation efficiency occurring in an argon matrix: acetaldehyde demonstrates a rather high yield of methyl radicals, while only trace amounts of these species were found for acetone and acetonitrile. Both effects were explained by the mechanism of “hot” ionic fragmentation resulting in the cleavage of the CH_3_-C bond in the corresponding excited radical cations, for example:

(CH_3_CHO^+**•**^)* → HCO^+^ + CH_3_**^•^** (11)

(CH_3_CH_2_OH^+**•**^)* → CH_2_OH^+^ + CH_3_**^•^** (12)

In a number of cases (including both astrochemically relevant molecules and large organic molecules), this mechanism has been directly confirmed by the EPR studies in matrices containing electron scavengers [13,38]. It should be noted that a similar mechanism was previously proposed for the formation of methyl radicals under the radiolysis of various hydrocarbons in solid argon [18,26]. Regarding the matrix effect, it is just what is expected from the general scheme outlined in Section 2. Indeed, turning from argon to xenon results in lower excess energy of the radical cation produced by the positive hole transfer (due to lowering the matrix IE) and higher efficiency of intermolecular relaxation (due to increasing the matrix polarizability). Both these effects should lead to suppression of the “hot” ionic fragmentation. The implication of this basic medium effect for the typical astrochemically relevant molecular ices is not fully clear, but one may speculate that the fragmentation would more efficiently occur in the CO-based ices (CO has high IE and relatively low polarizability, and both characteristics are comparable to those of krypton) than in the CO_2_-based, and, particularly, H_2_O-based ices.

The explanation of the effect of molecular structure is less straightforward, but it is apparently attributed to the effect of intramolecular relaxation [38]. In this connection, it is worth noting that unsaturated and aromatic radical cations with sufficiently high excess energy produced in an argon matrix are remarkably stable to “hot” fragmentation [41,42,43].

The mechanism of formation of methyl radicals from methanol implies that the C-O bond cleavage is less obvious. We may only note that the yield of this process is relatively low in comparison with the dehydrogenation of the CH_3_OH molecule [36]. Generally, we may conclude that the “hot” ionic fragmentation most probably plays a major role in the formation of methyl radicals from various organic molecules under their irradiation in rigid, inert media, but other mechanisms of their production cannot also be excluded.

Considering the possible reactions of methyl radicals, in view of their potentiality for cold synthetic astrochemistry, it can be first noticed that we did not observe a clear sign of diffusion and decay of these species in solid argon below 30 K and solid xenon below 50 K when the matrices are rigid enough. Thus, the diffusion of methyl radicals and their bulk recombination with other radical species in the bulk of rigid molecular ices at cryogenic temperatures is highly questionable, but such reactions may occur upon warming the irradiated ices or diffusion at the ice grain surface. It should be mentioned that annealing of the VUV photolyzed CH_3_OH/Ar system up to high temperatures (removal of argon matrix) results in the formation of several recombination products detected by IR spectroscopy and mass spectrometry [11]. Meanwhile, it is worth noting that at least one reaction of the methyl radical was recently directly observed in our studies in a noble gas matrix at an extremely low temperature [39]. In that work, the methyl radicals were generated by photolysis of acetyl (CH_3_CO**^•^**) radicals produced from acetaldehyde. Dissociation of CH_3_CO**^•^** under the action of visible light yields a CH_3_**^•^** ⋯CO pair located within the same matrix cage, and it was found that the reaction between these fragments led to the CH_3_CO**^•^** radical occurring in the timescale of tens of minutes in the dark, even at 5 K [39]. Most probably, this process involves tunneling. Interestingly, no reaction was found in other noble gas matrices under similar conditions, which provokes theoretical studies on the reaction dynamics with explicit account of the matrix environment. Generally speaking, this reaction may be significant for the radiation-induced cold processes in the CO-rich ices.

#### 3.1.2. C_2_ Hydrocarbon Radicals

The family of C_2_ hydrocarbon radicals includes ethynyl (C_2_H**^•^**), vinyl (C_2_H_3_**^•^**), and ethyl (C_2_H_5_**^•^**) radicals. In this row, only C_2_H**^•^** was reliably detected in the ISM [44]; however, all these species may give an important contribution to the ice chemistry leading to the formation of COMs in the ISM, comets, and outer planets of the Solar system. It is worth noting that the corresponding parent C_2_ hydrocarbons (acetylene, ethylene, and ethane) were found in comets [45] and at Titan (a satellite of Saturn) [46], which stimulated extensive model studies of the radiation-induced processing of neat C_2_-hydrocarbon ices [47,48,49,50,51,52,53]. In our recent study [54], it was directly shown that the above-mentioned C_2_ radicals result as important intermediates upon irradiation of the corresponding matrix-isolated hydrocarbon molecules (acetylene, ethylene, and ethane).

The ethynyl radical generated by different methods in the noble gas matrices was previously extensively characterized by vibrational (IR) and electronic absorption (UV) spectroscopy (see Ref. [12] for compilation of the results) and by EPR spectroscopy in an argon matrix [55]. The FTIR studies [56] showed that this radical is the only detectable primary intermediate of the radiation-induced transformations of acetylene molecules in all the noble gas matrices, which corresponds to a simple dissociation reaction:

C_2_H_2_* → C_2_H**^•^** + H**^•^** (13)

Our earlier combined EPR/FTIR studies on the radiation-induced chemistry in the C_2_H_2_/Xe system [56] directly demonstrated that the amounts of trapped hydrogen and ethynyl radicals were nearly balanced at low-absorbed doses. The C_2_ molecule (which becomes IR active and hence detectable by FTIR only in a xenon matrix due to strong complexation with a Xe atom [57]) is formed with a pronounced induction period (at high absorbed doses) due to a secondary process:

(C_2_H**^•^**)* → C_2_ + H**^•^** (14)

It should be noted that trace amounts of vinyl radicals were also detected by EPR in a xenon matrix, most probably as a result of the addition of “hot” hydrogen atoms formed in Reaction (13) to acetylene molecules:

C_2_H_2_ + H**^•^** → C_2_H_3_**^•^** (15)

However, this reaction mainly occurs upon warming the irradiated samples (see below).

Concerning the spectroscopic characterization of the ethynyl radicals, one could notice that the detection of this species in a xenon matrix by both FTIR and EPR spectroscopy is relatively low sensitive. Indeed, the principal IR feature corresponding to the CC stretching mode (at 1852 cm^−1^ in xenon) is weak and broad, partially due to strong interaction with a polarizable xenon matrix, and the characteristic doublet in the EPR spectrum is strongly broadened due to magnetic interaction with the matrix nuclei with non-zero spin (^129^Xe and ^131^Xe). It was demonstrated that the latter effect is removed in the case of a monoisotopic ^136^Xe matrix (I = 0) [35], which may be generally helpful in certain cases for the EPR measurements in xenon. On the other hand, both vibrational features and EPR signals are much clearer seen in argon because of its low polarizability and absence of magnetic nuclei. Generally speaking, such kinds of matrix effects on the radical spectra should be taken into account upon consideration of the relative abundance of radicals produced in various media.

The ethynyl radical is expected to be reactive so that it can be an important intermediate in the radiation-induced chemistry in the acetylene containing complex cosmic ices. However, to our knowledge, there is no direct evidence for such reactions in the low-temperature matrices. Some of them may proceed through intermediate formations of radical–molecule complexes, which will be discussed in the next section.

Vinyl radical was previously characterized in solid noble gas matrices by FTIR spectroscopy [30] and EPR [58,59,60]. Its EPR spectra demonstrate specific restricted dynamics of C_2_H_3_**^•^** in a xenon matrix [35,61]. Regarding vibrational spectra, the most comprehensive study in neon was reported by Wu et al. [62], which revealed six fundamentals assigned to C_2_H_3_**^•^**. Meanwhile, the most intense feature corresponds to the mixed OPLA vibrations appearing around 900 cm^−1^. This absorption band can be clearly seen even at relatively low concentrations of vinyl radical in matrices [56,61]. As mentioned above, this species may be generated by the thermally induced reaction of hydrogen atoms with acetylene molecules (15) occurring upon annealing the irradiated C_2_H_2_/Ng matrices at 30–45 K, depending on the matrix [61] (it is worth noting that, in the cases of xenon and krypton, this reaction of mobile hydrogen atoms competes with the formation of unusual noble gas hydrides, HXeH, HXeCCH, and HKrCCH, which was the main focus in a number of studies on the C_2_H_2_/Ng systems [56,63,64]). As revealed by our recent FTIR spectroscopic study [54], vinyl radical is one of the principal products of the irradiation of C_2_H_4_/Ng systems with X-rays (previously effective formation of this radical from ethylene was found under VUV photolysis of the C_2_H_4_/Ne matrix [62]). However, in the case of ethylene, dissociation of the parent molecules occurs via two channels (radical and molecular), which yield vinyl radical and acetylene, respectively:

C_2_H_4_* → C_2_H_3_**^•^** + H**^•^** (16)

C_2_H_4_* → C_2_H_2_ + H_2_ (17)

It was found [53] that the branching ratio between Reactions (16) and (17) increased within the row Ar < Kr < Xe. In other words, it means that the vinyl radical is more efficiently produced in a more polarizable xenon matrix. Thus, one may expect that it should also be the case for ethylene embedded in molecular ices. The formation of ethynyl radical was also observed at high absorbed doses. Obviously, it originates from the dissociation of acetylene accumulated in the system under prolonged irradiation. Regarding the low-temperature reactions of vinyl radicals, so far, there is no direct experimental evidence of such processes in matrices or laboratory ices, but they may occur either thermally or photochemically.

Ethyl radical in a solid argon matrix was first characterized by EPR in an early study [65], while its IR spectra were obtained in argon [66,67,68] and *para*-hydrogen [69] matrices. It is worth noting that, in all these studies, the ethyl radicals were generated not from ethane but from other precursors. The EPR spectrum of ethyl radical exhibits a broadened anisotropic pattern due to protons of methyl and methylene group (superior resolution was obtained under in situ irradiation of liquid ethane, which made it possible to precisely determine the isotropic coupling constants [70]). The most intense absorption in the FTIR spectrum corresponds to the CCH_2_ umbrella mode (541 cm^−1^ in argon [66]). The FTIR studies on the radiolysis of matrix-isolated ethane [54] demonstrated that the most important primary process is dehydrogenation, which results in the formation of ethyl radical and ethylene:

C_2_H_6_* → C_2_H_5_**^•^** + H**^•^** (18)

C_2_H_6_* → C_2_H_4_ + H_2_ (19)

The matrix trend is similar to that observed for ethylene: relative yield of ethyl radical increases in the row Ar < Kr < Xe, but the effect is much more pronounced in the case of ethane [54]. The matrix effect can be explained by the involvement of “hot” reaction channels, i.e., fragmentation of “hot” ethane radical cation:

(C_2_H_6_^+**•**^)* → C_2_H_4_^+**•**^ + H_2_ (20)

followed by recombination of the resulting ethylene radical cations with electrons. Indeed, in accordance with the above-considered general scheme, the relative efficiency of “hot” fragmentation (20), finally given the ethylene molecule, is expected to be much lower in xenon because of its smaller IE (close to ethane) and higher polarizability. An alternative (or additional) explanation may be concerned with the high efficiency of intersystem crossing in xenon (external heavy atom effect), which leads to a higher population of triplet excited states, presumably responsible for the formation of ethyl radicals in Reaction (18) [54]. In addition to Reactions (18) and (19), in the case of ethane, we also revealed evidence for the formation of vinyl radicals and acetylene molecules [54]. These products, most probably, result from secondary processes of ethylene dissociation (16) and (17), so their contribution increases at high absorbed doses. However, the formation of both vinyl radicals and acetylene is observed already at low-absorbed doses (particularly in argon), which may imply that the ethylene molecules are partially formed in a vibrationally excited state and undergo prompt dissociation, looking as a quasi-single-step process. Obviously, the efficiency of the energy dissipation to matrix lattice increases from argon to krypton and xenon, so the probability of prompt formation of vinyl radicals and acetylene from ethane becomes lower.

Regarding the implications of the matrix-isolation studies for molecular ices, one may expect that the radiation-induced formation of ethyl radicals should represent an important or predominating primary reaction channel in various ices (the “hot” fragmentation should be suppressed, with possible exception for a low polarizable CO-based ices). In this connection, it is worth considering available data on the radiolysis of neat solid ethane at cryogenic temperatures. As was shown in an early EPR study [71], ethyl radical is the principal primary paramagnetic product of gamma-radiolysis of solid ethane. Our FTIR study [54] demonstrated that the basic products resulting from X-ray irradiation of ethane ice in both crystalline and amorphous states at 5 K are ethyl radical and ethylene. Remarkably, the relative yield of the former species is much higher than that for matrix-isolated ethane irradiated under similar conditions. Other observable products are butane and methane. While butane definitely originates from bimolecular reactions [72], the observation of methane is somewhat surprising because this product of the C-C bond cleavage was observed only in trace amounts under the condition of matrix isolation. In any case, the C-H bond cleavage strongly predominates in ethane ice phases (similar to matrices), and methyl radical was not found. Thus, ethyl radical should be the key reactive intermediate produced from ethane upon irradiation in various icy media.

Summarizing the present knowledge on the radiation-induced transformations of C_2_ hydrocarbons in cryogenic media, one may conclude that the radicals produced by single C-H bond rupture are the key primary reactive intermediates in most cases and they could be the building blocks in synthetic cold astrochemistry occurring upon warming the corresponding ices. The loss of two or more hydrogen atoms becomes more important at high absorbed doses; however, it may also occur as a quasi-single-step process in low-polarizable media, where the dissipation of excess energy to a matrix is relatively inefficient.

#### 3.1.3. Radicals Produced from Aromatic Hydrocarbons

Simple aromatic hydrocarbons are supposed to be the precursors of PAH molecules, which may be formed in space in different ways [73,74]. Benzene molecule was detected in the ISM some twenty years ago [75], and the impact of ion irradiation on the neat benzene ices was previously studied with astrochemical motivation [76,77].

Independent of the detailed mechanism, the way from benzene to PAHs implies activation of the C_6_H_6_ molecule retaining its molecular skeleton. One of the most common possible routes is concerned with the primary formation of phenyl (C_6_H_5_**^•^**) radical. Even though direct astronomical observation of this radical is lacking, it is supposed to play an important role in astrochemistry (see Ref. [77] and references therein). Phenyl radical was previously obtained and characterized under the conditions of matrix isolation by IR spectroscopy [30] and EPR [78]. It should be noted that in all these studies, it was generated from some suitable precursors (benzene derivatives) and not directly from benzene. Meanwhile, the UV photolysis of matrix-isolated benzene in solid argon [79,80] and *para*-hydrogen [81] revealed the formation of various benzene isomers and a minor production of phenyl radical. Using a combination of FTIR and EPR spectroscopy, we have demonstrated [82] that irradiation of benzene in a solid xenon matrix with fast electrons provides a high yield of dissociation of the benzene molecule to phenyl radical and hydrogen atom:

C_6_H_6_* → C_6_H_5_**^•^** + H**^•^** (21)

Regarding the spectroscopic aspects, it is worth noting that the EPR signal of phenyl radical is anisotropically broadened and may overlap with the signals from other species in complex systems, while the most intense IR absorption band corresponding to the CH deformation mode (703 cm^−1^ [82] in Xe, 706 cm^−1^ in Ar [30]) is strong enough and rather characteristic, so it can be used for unambiguous detection of this radical (to note, six fundamentals of the C_6_H_5_**^•^** radical were observed in the irradiated C_6_H_6_/Xe system [82]). A more recent FTIR study of the effect of X-ray irradiation on solid of C_6_H_6_/Ng systems (Ng = Ar, Kr, Xe) at 6 K [83] demonstrated that dissociation (21) appears to be the major primary reaction channel for benzene in all the matrices, but the competition with isomerization to fulvene should be taken into account in krypton and, particularly, in argon. Based on consideration of matrix effects and comparison with photolysis, it was proposed [83] that dissociation (21) most probably occurs from high triplet excited states (or from vibrationally “hot” T_1_ state). This finding is important because it suggests that the formation of phenyl radical from benzene in rigid cryogenic media (including astrochemically relevant ices) most probably would require indirect activation by ionizing radiation rather than direct photoexcitation.

Cyclohexadienyl (c-C_6_H_7_**^•^**) radical is another benzene-related species that may be potentially important for astrochemistry [81]. It was explicitly shown [82] that this radical is effectively formed upon annealing of the irradiated matrix-isolated C_6_H_6_/Xe system due to the reaction of the thermally mobilized hydrogen atoms:

C_6_H_6_ + H**^•^** → c-C_6_H_7_**^•^** (22)

While the characteristic EPR spectrum of this radical exhibiting unusually large hyperfine coupling with two protons (~4.7 mT) was known for several decades [84], the IR spectrum was first characterized on the basis of comparison with EPR data and quantum-chemical calculations in our work [82]. It is worth noting that at least six vibrational fundamentals of this radical were assigned in a solid xenon matrix [82] and confirmed in a later FTIR study using a *para*-hydrogen matrix [81].

The formation of a second aromatic ring (finally leading to PAH) may also involve the intermediates produced from alkylbenzenes. The low-temperature radiation-induced transformations of such molecules are poorly studied, and they may be potentially related to astrochemistry. Recently, it was shown [85] that irradiation of a solid C_6_H_5_CH_3_/Ng system results in the efficient formation of benzyl (C_6_H_5_CH_2_**^•^**) radical:

C_6_H_5_CH_3_* → C_6_H_5_CH_2_**^•^** + H**^•^** (23)

The FTIR spectrum of benzyl radical generated by pyrolysis of suitable precursors was thoroughly characterized previously in an argon matrix [86], and on the basis of comparison with these results, we were able to assign ten fundamentals of this species in the irradiated C_6_H_5_CH_3_/Xe system (and, at least, eight lines in argon and krypton matrices) [85]. Similar to the case of benzene, the relative yield of the C-H bond dissociation (23) increases in the row Ar < Kr < Xe (in the case of toluene, the main competing reaction channel is isomerization to 5-methylene-1,3-cyclohexadiene [85]). Based on the matrix effect, it was suggested [85] that benzyl radical mainly originates from the high triplet excited states (T_n_), but it may also be formed from a vibrationally hot ground state (S_0_).

Generally speaking, the elucidation of the nature of radical species resulting from irradiation of aromatic hydrocarbons in cryogenic media and their possible role in astrochemistry deserves further investigation. It is worth noting that these species may be important not only for the formation of PAH but also for cold synthesis of functional benzene derivatives. One such molecule, benzonitrile (C_6_H_5_CN), was recently directly observed in the ISM [87].

### 3.2. C,O-Containing Radicals

The small radicals simultaneously containing C and O atoms are potentially very significant intermediates in prebiotic astrochemistry. A number of such species were directly observed in the ISM, and their spectra were previously assigned by matrix isolation studies. Meanwhile, recent studies using radiolysis or VUV photolysis in matrices revealed new important features concerning the formation mechanism, structure, and dynamics of these radicals.

#### 3.2.1. Formyl and Hydrocarboxyl Radicals

Formyl (HCO**^•^**) radical was detected in the ISM in 1986 [88]. In addition to astrochemistry, it is a relevant intermediate in combustion, atmospheric chemistry, and other fields. Under laboratory conditions, this triatomic radical was characterized in cryogenic media some sixty years ago using IR spectroscopy [89] and EPR [90] (the compilation of spectroscopic data is available in Refs. [30,65] for IR and EPR spectroscopy, respectively). The EPR spectra of formyl radicals reveal characteristic anisotropic doublet with extremely large hyperfine splitting (~13 mT, matrix dependent), which usually facilitates its detection even in complex systems. The IR spectra of HCO**^•^** in matrices show all three fundamental vibrational features, the CO stretching (1863 cm^−1^ in Ar) being the strongest one [30]. While the vibrational frequencies of this species are experimentally available with sufficient precision, the knowledge of absolute IR intensities (important for various applications, including laboratory astrochemistry) relied on theoretical calculations. In a recent study [91], we first experimentally determined the corresponding values for all three absorption bands observed in an argon matrix (this is probably the first example of comprehensive determination of the IR intensities for a radical containing more than two atoms to note). The results obtained demonstrated relatively good agreement with modern theory for the CO stretching and CH stretching bands but a rather large discrepancy for the HCO bending mode, which is a challenge for further efforts in this field.

The formyl radical in various matrices is often obtained by the reaction of hydrogen atoms with CO molecules [91] and references therein:

H**^•^** + CO → HCO**^•^** (24)

As revealed in our studies, it is also produced in the radiation-induced processes from various astrochemically relevant organic compounds, namely, methanol [37], ethanol [37], and acetaldehyde [39]. The formation of this radical was also found under VUV photolysis of methanol in solid argon using a combination of EPR and IR spectroscopy [11]. In the case of methanol, the HCO**^•^** radical results from “deep dehydrogenation” (loss of three hydrogen atoms occurring as a quasi-single-step process). It was suggested [37] that such a process involves the intermediate formation of vibrationally hot formaldehyde molecule, which is not stabilized in a matrix but undergoes prompt dissociation:

CH_3_OH* →[CH_2_O]*(+H_2_) → HCO**^•^** + H**^•^** (25)

The observed matrix trend is consistent with this suggestion: the efficiency of Reaction (25) increases and decreases in the row Ar > Kr > Xe, which can be explained by the increasing probability of excess energy dissipation (intermolecular vibrational cooling) in more polarizable matrices. For the molecules containing two carbon atoms, the formation of formyl radical implies the C-C bond cleavage (skeleton fragmentation, which may occur from both “hot” radical cations and neutral excited states [37,39]). The matrix effect (higher efficiency of this process in argon) suggests a similar explanation as for the C_2_ hydrocarbons considered in Section 3.1.2, which is based on more efficient energy dissipation in matrices with higher polarizability and stronger interaction.

Hydrocarboxyl radical (HOCO**^•^**, also sometimes referred to as carboxyl radical) has not yet been detected in the ISM, but its involvement in the formation of COMs and simple biomolecules is discussed in a number of works. In particular, recently, it was shown that the HOCO**^•^** radical can be produced by UV photolysis of simple amino acids in a para-hydrogen matrix [92], which justifies it as a tracer of amino acids and may imply its involvement in their synthesis. The EPR spectrum of this carbon-centered radical is not characteristic because of small proton hyperfine coupling, and its unambiguous assignment is difficult, especially if it is present as an admixture to other radical species (the EPR detection of HOCO**^•^** observed under such conditions was recently reported under VUV photolysis of methanol in an Ar matrix [11]). In contrast, the IR signatures of the hydrocarboxyl radical in matrices are quite clear (the compilation of the results is available [30]. The strongest feature corresponding to the CO stretching appears at 1843.6 cm^−1^ in argon, with reasonable shifts in other matrices). Using FTIR spectroscopy, we have shown that, basically, the radiation-induced formation of this intermediate in cryogenic matrices may occur through both synthetic and decomposition paths. The former way is based on the reaction within the radical–molecule complex:

HO**^•^⋯**CO → HOCO**^•^** (26)

The radical–molecule complex (to be discussed in more detail in the next section) can be produced either from the H_2_O**⋯**CO molecular complex (by VUV photolysis [93] or radiolysis [94]) or from H_2_O**⋯**CO_2_ complex (by radiolysis [95], in the latter case, intermediate formation of the radical-complex was not shown directly).

The decomposition pathway was first demonstrated for the X-ray radiolysis of formic acid in solid noble gas matrices [96]:

HCOOH* → HOCO**^•^** + H**^•^** (27)

It was suggested [96] that Reaction (27) occurred from triplet excited states populated through ion–electron recombination. It is worth noting that the HOCO**^•^** radical may stabilize in the form of cis- and trans-conformers at low temperatures. The low-temperature internal dynamics of these species were carefully studied in different cryogenic matrices [97], which revealed that the conversion between the two conformers may occur in the dark (via tunneling mechanism) or under selective IR pumping. The investigation of such transformations may be generally significant to better understand the nature of stereoselectivity in cold astrochemical processes.

The potential role of the hydrocarboxyl radical as an intermediate in synthetic astrochemistry was demonstrated in our recent study on the radiation-induced synthesis of formic acid in solid noble gas matrices [94].

#### 3.2.2. Hydroxyalkyl and Alkoxy Radicals

Alpha-hydroxyalkyl (RCHOH**^•^**) and alkoxy (RCH_2_O**^•^**) radicals are isomeric species, which correspond to the hydrogen atom detachment from primary alcohols. Among these species, the two first representatives (R = H and R = CH_3_) are mostly relevant to astrochemistry because they can be produced from methanol and ethanol detected in the ISM [98,99]. The CH_2_OH**^•^** and CH_3_CHOH**^•^** produced from irradiation of solid methanol and ethanol, respectively, at 77 K, were characterized by EPR several decades ago [100]. For both alcohols, these are the principal radical products of radiolysis under the above-mentioned conditions. However, their EPR detection under VUV photolysis or radiolysis of isolated alcohol molecules in the noble gas matrices was reported only recently [11,37]. The IR spectrum of CH_2_OH**^•^** in solid argon and nitrogen matrices was first reported by Jacox and Milligan in 1973 [101], whereas the vibrational features of the CH_3_CHOH**^•^** radical is still experimentally unavailable from matrix or gas-phase studies.

The studies on radiation-induced transformations of methanol molecules at cryogenic temperatures are vitally important for astrochemistry because CH_3_OH is believed to be one of the key simple organic molecules detected in the ISM [98]. The formation of the CH_2_OH**^•^** radical under X-ray irradiation of methanol isolated in different noble gas matrices (from Ne to Xe) was confirmed by FTIR studies [36]. It was also observed by both FTIR and EPR spectroscopy under VUV photolysis of the CH_3_OH/Ar system [11]. Ethanol is the second important alcohol molecule in astrochemistry and cold prebiotic evolution, and its first astronomical observation was reported almost fifty years ago [99]. The hydroxyethyl radical is probably an important intermediate in ethanol-related cold space chemistry, even though its direct observation in space is still pending. To the best of our knowledge, the first EPR identification of CH_3_CHOH**^•^** produced by X-ray irradiation of isolated ethanol molecules in cryogenic media was reported in our recent study [37]. Remarkably, this radical was found to be the key radical species produced in solid xenon but not in an argon matrix. The effect was explained by the predominating contribution of “hot” skeleton fragmentation in the case of argon, as discussed above. It should be noted that the CH_3_CHOH**^•^** radical observed in solid xenon demonstrates a frozen conformation with a non-rotating methyl group at 7 K, while the motional averaging is observed at ca. 50–60 K [37]. It is worth noting that reliable EPR detection of the CH_3_CHOH**^•^** radical in solid xenon can provide a reference basis for the assignment of its vibrational features in the IR spectra, as preliminary data were published quite recently [102].

The alkoxy radicals produced from simple alcohols (CH_3_O**^•^** and CH_3_CH_2_O**^•^**) present a real challenge from both spectroscopic and kinetic viewpoints. The methoxy radical (CH_3_O**^•^**) has been detected in the ISM [103], while its analog, ethoxy radical, was not yet directly observed. The EPR observation of alkoxy radicals in solid matrices is non-trivial because of the specific electronic structure and low stability of these species, even at low temperatures. The orbital degeneracy of the ground state results in very large g anisotropy (due to the significant contribution of orbital momentum) and severe signal broadening in macroscopically disordered media. The orbital degeneracy may be partially lifted in strongly interacting media, and this explains the observation of the methoxy radical in solid crystalline methanol at 4.2 K [104]. It is characterized by a remarkably high g_xx_ component of the g tensor and large coupling constants with β-protons (as compared to C-centered radicals). However, the reliable EPR detection of this radical (as well as the ethoxy radical) in low-interacting media, including noble gas matrices, is questionable. The spectrum observed after radiolysis of methylal (CH_3_OCH_2_OCH_3_) in solid argon was assigned to the complex of CH_3_O**^•^** with the CH_2_OCH_3_^+^ cation [105], in which the orbital degeneracy is removed due to strong local interaction with the cationic center. The IR spectrum of the methoxy radical in matrices was also a subject of discussion. A comprehensive characterization was made in a para-hydrogen matrix, where methoxy radical was obtained by UV photolysis of methoxy nitrite [106]. Later on, Gutiérrez-Quintanilla et al. reported the IR spectrum of this radical produced by VUV photolysis of methanol in an argon matrix [11] (it is worth noting that no sign of the methoxy radical was seen in the EPR spectrum under similar conditions [11], probably due to the spectroscopic problem described above). Regarding the kinetic issue with the methoxy radical, it concerns its intrinsic stability in low-temperature environments. From a thermodynamic point of view, the CH_3_O**^•^** radical is less favorable than its more stable isomer (CH_2_OH**^•^**), which suggests its possible intramolecular conversion:

CH_3_O**^•^** → CH_2_OH**^•^** (28)

Meanwhile, the computed barrier for such transformation was found to be very high [107], which makes the possibility of such conversion questionable at cryogenic temperatures or, at least, implies the predominating contribution of tunneling [36]. At this point, it should be noted that a relatively fast transformation of methoxy radical to its more favorable isomer was observed in both methanol at 4.2 K [104] and *para*-hydrogen matrix [106]. This observation generally justifies the involvement of tunneling. However, Gutiérrez-Quintanilla et al. did not find any measurable decay of the methoxy radical in an argon matrix [11], which suggests that the transformation observed in molecular solids may be not due to intramolecular rearrangement, but due to intermolecular hydrogen abstraction from the neighboring matrix molecules (CH_3_OH or *para*-H_2_). On the other hand, careful inspection did not allow us to find any spectroscopic features that could be assigned to the CH_3_O**^•^** radical after X-ray irradiation of the CH_3_OH/Ng systems (Ng = Ne, Ar, Kr, Xe) at 6 K [36]. Nevertheless, the experiments with deuterated methanol isotopologues [36] suggest that the methoxy radical should be formed in a primary process, but it is not stabilized in the noble gas matrix, which formally can be explained by prompt intramolecular conversion:

CH_3_O* → [CH_3_O**^•^**] → CH_2_OH**^•^** (29)

The discrepancy between the data obtained for direct (VUV [11]) and indirect (X-rays [36]) activation of methanol molecules in matrices may be tentatively explained by a different mechanism of their formation or different precursor states involved, for example, Reaction (29) may occur through the formation of vibrationally “hot” methoxy radical. The strong matrix effect on the tunneling isomerization may be considered. Additionally, it should be noted that the methoxy radical is a relatively poor IR absorber, so its FTIR detection may be problematic if it occurs in minor or trace amounts.

Regarding ethoxy (CH_3_CH_2_O**^•^**) radical, apparently, there are no experimental reports on its spectroscopic characterization. General features of these species (orbital degeneracy, large g anisotropy, and large hyperfine coupling constants with the β-protons of methylene group) should be similar to those for CH_3_O**^•^** radical. Our recent EPR study [37] did not provide any evidence for the stabilization of these radicals after irradiation of isolated ethanol molecules in solid argon and xenon. Meanwhile, similar to the case of methanol, there is a strong indirect indication of their involvement in the primary processes from the isotopic labeling studies. Again, one may consider a two-stage process leading to α-hydroxyethyl radical observed in the experiment:

CH_3_CH_2_OH* → [CH_3_CH_2_O**^•^**] → CH_3_CHOH**^•^** (30)

It should be stressed that both hydroxyalkyl and alkoxy radicals may participate in the recombination processes upon warming the irradiated ices. The formation of various complex molecules resulting from recombination was directly demonstrated after annealing the VUV-photolyzed CH_3_OH/Ar system when the argon matrix was removed [11].

#### 3.2.3. Acetyl and More Complex Radicals

The acetyl radical (CH_3_CO**^•^**) is a potential constituent that can be used for building various COMs in space environments, while its direct astronomical detection is still pending. The provisional EPR signature of acetyl radical in irradiated solid molecular media (a broad unresolved singlet signal) was reported in the early period [63], and the IR spectrum in a solid argon matrix obtained by reaction of F atoms with acetaldehyde was presented by Jacox [108]. Our studies on the radiolysis of acetaldehyde in solid noble gas media revealed the formation of this radical, as confirmed by both IR and EPR data [37,38]. This observation seems to be important for astrochemistry since acetaldehyde was found in the ISM [44]. The matrix effect (increasing production of acetyl radicals in xenon in comparison with argon) is consistent with the mechanism suggesting the generation of the CH_3_CO**^•^** radicals from triplet excited states [39]:

CH_3_CHO* → CH_3_CO**^•^** + H**^•^** (31)

On the other hand, the EPR experiments in xenon matrices containing electron scavengers [38] provided evidence for prompt formation of the acetyl radical from the primary acetaldehyde radical cation, which was interpreted as a result of deprotonation to matrix:

CH_3_CHO^+**•**^ + 2Xe → CH_3_CO^+^ + Xe_2_H^+^ (32)

Meanwhile, detailed consideration of the EPR and IR spectroscopic results [39] made us assume that the reaction observed in xenon is, most probably, a xenon-catalyzed isomerization of the radical cation:

CH_3_CHO^+**•**^ (Xe) → CH_3_CO**^•^**(H^+^) (33)

The product of Reaction (33) is protonated acetyl radical, a carbon-centered species, which may be undistinguishable from CH_3_CO**^•^** by EPR spectroscopy. It is worth noting that such kind of medium catalysis may also occur for the radical cations produced in icy molecular matrices.

The acetyl radical is a photosensitive species that undergoes efficient dissociation under the action of visible light:

CH_3_CO**^•^** + hν → [CH_3_**^•^** + CO] (34)

As mentioned in Section 3.1.1, if the products of Reaction (34) stay in the same matrix cage, they can undergo a dark reaction restoring the acetyl radical, or, alternatively, they can diffuse (even at low temperatures) and enter other chemical reactions.

A carbon-centered CH_2_CHO**^•^** radical is an isomer of acetyl radical produced by hydrogen atom detachment from the methyl group. It was characterized by IR spectroscopy in solid argon [108], whereas the unequivocal EPR identification of this species is still lacking. We did not find conclusive evidence for the formation of this radical under X-ray irradiation of the CH_3_CHO/Ng systems [39], although the EPR studies on the same system may tentatively imply its occurrence [38]. The possible role of CH_2_CHO**^•^** in cold astrochemistry is unclear.

The radicals produced from carboxylic acids (RCOOH) may play in important role in the astrochemical synthesis of amino acids. The simplest species of this kind is CH_2_COOH**^•^** radical related to the transformations of acetic acid, which was found in the ISM some twenty years ago [109]. A triplet signal observed in the EPR spectra of acetic acid irradiated at 77 K was tentatively assigned to this radical several decades ago [65], while its IR spectrum in a *para*-hydrogen matrix was reported only quite recently [110]. Further studies on the spectroscopy and reactions of this radical present a considerable interest in understanding the mechanisms of formation of COMs (and, in particular, basic amino acids) in space environments.

Among the astrochemically relevant radicals containing two carbon atoms and an oxygen atom, one could also mention the ketenyl (HCCO**^•^**) radical, which was detected in space relatively recently [111]. This radical was observed by FTIR spectroscopy after irradiation of acetaldehyde in solid noble gas matrices [39] or as a result of radiation-induced transformations of C_2_H_2_/H_2_O/Ng systems [112], whereas its unambiguous EPR characterization is still pending. In both cases, the ketenyl radical occurs as a relatively minor product, most probably resulting from ketene (H_2_CCO). The latter molecule was found in the ISM long ago [98], and it can be an important species in cold astrochemistry due to its high and diverse reactivity.

The open-shell species containing three carbon atoms and an oxygen atom will be considered below in connection with the radiation-induced evolution of the C_2_H_2_/CO/Ng systems.

### 3.3. C,N-Containing Radicals

Nitrogen is a vitally important atom in prebiotic evolution, so the formation of the reactive open-shell species simultaneously containing C and N atoms presents considerable interest in astrochemistry.

#### 3.3.1. The Radicals Related to HCN

The simplest radical of this kind is cyanogen (CN**^•^**), known from the earliest astronomical observations [23]. The characterization of CN**^•^** under the conditions of matrix isolation was performed by both EPR [113] and IR spectroscopy [114,115]. Nevertheless, detailed information on its spectroscopic properties and dynamics in different media was limited for a long time. It is worth noting that the CN**^•^** fundamental frequency is rather specific and characteristic (the absorption band at 2240–2244 cm^−1^ was observed in various matrices [116,117,118]). However, it is a poor IR absorber, which may preclude its detection if it occurs in low concentration. Most commonly, the cyanogen radical may be generated from HCN, one of the most important triatomic molecules in space. This radical can also be produced by the photolysis of other molecules of astrochemical interest, such as cyanoacetylene and dicyanoacetylene to note [119]. Using a combination of FTIR and EPR spectroscopy, we have shown that the X-ray irradiation of the HCN/Ng (Ng = Ne, Ar, Kr, or Xe) deposited mixtures at 7 K leads to efficient dissociation of hydrogen cyanide yielding CN**^•^** and H**^•^** atoms [116]. It is worth mentioning that the CN**^•^** radical is non-rotating in solid argon and krypton at 7 K but exhibits rotation in the EPR timescale and rotational structure in the IR spectra in a more spacious Xe matrix at the same temperature [116]. The issue of rotation of this radical in molecular icy media may be important for its preferential reactivity if it is formed in the same cage with some other radical [117]. The reactions of cyanogen radical may be significant for the formation of various COMs containing nitrogen atoms.

Two other radicals observed in the irradiated HCN/Ng systems are isomeric hydrogenated species, H_2_CN**^•^** and HCNH**^•^** [116]. The former radical was found in the ISM [118], while the latter species was not yet detected. In the case of the matrix isolation experiment, both radicals mainly result from an addition of the thermally mobilized hydrogen atoms to HCN:

HCN + H**^•^** → H_2_CN**^•^** (35)

HCN + H**^•^** → HCNH**^•^** (36)

The branching ratio between Reactions (35) and (36) in solid krypton was determined to be ca. 10 [116], which is in qualitative agreement with both thermodynamic and kinetic considerations [120]. It was concluded that the trans-conformer of HCNH**^•^** was formed in Reaction (36) to note [116]. In addition, it was found [116] that the HCNH**^•^** radical was selectively sensitive to photolysis with visible light (λ = 460–490 nm). This finding might explain the non-detection of this radical in astronomical observations.

#### 3.3.2. The Radicals Produced from Acetonitrile

Acetonitrile (CH_3_CN) is an important small organic molecule occurring in the ISM [98]. We have found that irradiation of the CH_3_CN/Ng deposited mixtures at 5–7 K results in the formation of a variety of products, including a number of open-shell species [40]. The isomeric CH_2_CN**^•^** and CH_2_NC**^•^** radicals were mainly observed by FTIR spectroscopy and relatively low- and moderate-absorbed doses [40] (the vibrational features of both these species are known from previous studies. See Ref. [12] for compilation). The latter radical (CH_2_NC**^•^**) probably originates from iso-acetonitrine (CH_3_NC) produced in a primary process (the CH_3_NC molecule was also directly observed in the ISM to note [121]). No pronounced matrix effect was found in the formation of the above-considered radical species.

The irradiation of the CH_3_CN/Ng systems to high absorbed doses results in the occurrence of secondary dehydrogenation processes leading to the formation of isomeric CCN**^•^** and CNC**^•^** radicals [122]:

(CH_2_CN**^•^**)* → CCN**^•^** + H_2_/2H**^•^** (37)

(CH_2_NC**^•^**)* → CNC**^•^** + H_2_/2H**^•^** (38)

The identification of these radicals [122] was made by FTIR spectroscopy on the basis of comparison with the known literature data [30] and confirmed by the absence of a deuterium substitution effect (the same absorption bands appeared for both CH_3_CN and CD_3_CN, which proves the absence of H atoms in their structure). It is worth noting that reliable EPR detection of CCN**^•^** and CNC**^•^** is still lacking.

It is unclear whether Reactions (37) and (38) include the formation of CHCN and CHNC species as intermediates, but, in any case, a quasi-single-step deep dehydrogenation (observed for methanol and some other molecules considered above) was not found, i.e., the CCN**^•^** and CNC**^•^** radicals appear from acetonitrile with a pronounced induction period.

An interesting feature of these two radicals is concerned with their efficient and partially reversible interconversion under the action of light. It was found [122] that photolysis with visible light (λ = 460–470 nm) results in selective and complete conversion of CCN**^•^** to CNC**^•^**, whereas subsequent photolysis with the UV-light (λ = 254 nm) leads to partial recovery of the CCN**^•^** radical, finally resulting in a photo-stationary state. Regarding the astrochemical implications, one may note that the CCN**^•^** radical has been astronomically detected [123], while its isomer was not yet found. The photochromism described above may be important for the fate of these radicals in space objects.

#### 3.3.3. C,N,O-Containing Radicals

The radicals simultaneously containing C,N, and O atoms are of particular interest for understanding the mechanisms leading to biologically important COMs in space. Direct spectroscopic information on such open-shell species in cryogenic matrices subjected to irradiation is rather limited. One could mention the work of Pettersson at al. [124] reporting the formation of H_2_NCO**^•^** and NCO**^•^** radicals under UV-photolysis of HNCO in a solid Xe matrix. In our recent FTIR spectroscopic study [125], it was shown that both these radicals were produced after X-ray irradiation of the NH_3_/CO/Ng (Ng = Ar, or Kr) deposited mixture at 5 K. It was concluded that the H_2_NCO**^•^** radical resulted from the NH_3_**⋯**CO intermolecular complex (the detailed mechanism is unclear), while the NCO**^•^** radical was formed though subsequent dehydrogenation of H_2_NCO**^•^** [125]. Synthetic processes of such kind may be generally important for understanding the mechanisms of the formation of COMs with amide moiety in mixed astrochemical ices subjected to ionizing radiation.

### 3.4. Radical Cations

The importance of ionized and, in particular, cationic species in astrochemistry was realized long ago. A number of simple cationic species were directly detected in space. Radical cations are open-shell species produced by ionization of closed-shell stable molecules. In theoretical simulations, they are often considered intermediates of the gas-phase astrochemical processes; however, direct manifestations of their involvement in solid-state astrochemistry are still limited. Simple inorganic astrochemically relevant radical cations (such as H_2_O^+**•**^, CO^+**•**^, NH_3_^+**•**^, and some other species) were characterized using EPR spectroscopy in neon matrices by Knight’s group [34]. The IR features of some of these species (usually produced by gas-phase discharge or photoionization followed by condensation) were also identified in solid neon and argon matrices [12,30]. Organic radical cations remained elusive for several decades because of their high reactivity, even at low temperatures. The breakthrough was achieved in the 1980s, and since that time, a large number of radical cations produced from complex organic molecules was studied by EPR in detail due to the invention of a convenient and simple laboratory technique based on their stabilization in halocarbon (Freon) matrices at moderately low temperatures (typically, 77 K) (several reviews are available [14,126,127,128]). An overview of EPR studies on moderate-size organic radical cations stabilized in noble gas matrices made in our laboratory was given elsewhere [14,17]. It should be noted, however, that small astrochemically important radical cations, in many cases, were not stabilized in halocarbon matrices for a number of reasons, such as high IE (comparable or even higher than that of the halocarbon host) and tendency to association in halocarbon solutions. Thus, the classical matrix isolation technique using solid noble gases is the only solution in this case. Among successful examples of using this technique, one could mention the identification of CH_4_^+**•**^ [129], CH_3_OH^+**•**^ [130], and CH_2_O^+**•**^ [131] in a neon matrix. It is worth noting that the CH_3_CHO^+**•**^ radical cation was characterized in neon [132], argon [38], and halocarbon [133] matrices. Among other astrochemically relevant aliphatic radical cations obtained in our previous studies in an argon matrix, one could mention CH_3_OCH_3_^+**•**^ [13] and CH_3_COCH_3_^+**•**^ [38], generated from dimethyl ether and acetone, respectively. Remarkably, none of the above-mentioned radical cations was reliably detected in solid krypton or xenon. As discussed elsewhere [14,15], most probably, the common reason is the relatively high polarizability of argon and krypton correlating with their proton affinity, which results in the occurrence of deprotonation of the highly acidic primary radical cations to a matrix (an example is Reaction (32)). In addition, the positive hole transfer from a xenon matrix may be inefficient (or even thermodynamically unfavorable) for the molecules with relatively high IE. It should be noted that deprotonation to a matrix is also highly probable for acidic radical cations produced in cosmic molecular ices, such as H_2_O- and CO_2_-based ices.

Among aromatic radical cations of potential astrochemical significance, a basically important benzene radical cation (C_6_H_6_^+**•**^) has been characterized by EPR in various noble gas matrices [41,43]. This Jahn–Teller active species is also of considerable interest to note from the viewpoint of general structural chemistry. Regarding the PAH radical cations, it is worth noting that such species should be particularly relevant to space chemistry because it is often assumed that the PAH molecules mainly exist in the ISM in the ionized form. However, detailed consideration of spectroscopic properties of PAH cations and the related diamagnetic protonated species radical cations is a special topic beyond the scope of this paper (a current overview of applications of matrix isolation to this issue may be found in a recent review [12]).

It should be noted that, in the case of radical cations, EPR spectroscopy remains the most important and widely used method for structural studies. The combination of this method with vibrational (IR) spectroscopy may bring significant new information. Some applications of such approach to radical cations were reported previously [18,19]. Nevertheless, the vibrational spectra of a number of small astrochemically important organic radical cations are still experimentally unknown. In this connection, attention should be paid to a recent example of the application of this combination to unravel astrochemical issues, which is related to the C_3_H_2_O^+**•**^ radical cations resulting from the radiation-induced transformations in the C_2_H_2_/CO/Ar system [134]. Using EPR and FTIR spectroscopy supported by ab initio calculations at the CCSD(T) level, it was shown that the E-HCCHCO^+**•**^ radical cation was stabilized as one of the key primary intermediates in the studied system:

[C_2_H_2_**⋯**CO]^+**•**^→ E-HCCHCO^+**•**^ (39)

Subsequent photolysis of this radical cation with visible light results in its transformation to the H_2_CCCO^+.^ structure:

*E*-HCCHCO^+**•**^ + hν → H_2_CCCO^+**•**^ (40)

This study has clearly demonstrated the benefit of combined spectroscopic studies: while the IR spectroscopy reveals the presence of carbonyl moiety affected by positive charge (strongly shifted CO stretching frequency), EPR data explicitly show the presence of anisotropic proton hyperfine coupling characteristic for the specific chemical structures. It is worth noting that both vibrational features and EPR parameters are in reasonable agreement with the quantum chemical calculations [134]. The theoretical analysis of the potential energy surface (PES) performed at the CCSD(T) level [134] demonstrates the whole proposed pathway for the evolution of the initial C_2_H_2_**⋯**CO complex after its ionization. It should be noted that the PES analysis for excited state dynamics in Reaction (40) performed later [135] is in agreement with the experimental observation [134]. Versatile implications of this study may be considered. On the one hand, the studied C_2_H_2_/CO system is believed to be one of the most important precursors for cold astrochemical synthesis of COMs, and the matrix isolation study [134] demonstrates the possible involvement of cationic pathways in its evolution. On the other hand, the strategy used in this work may be useful for obtaining and characterization of radical cations, which can hardly be produced by direct ionization of unstable neutral precursors in matrices.

Table 1 presents the list of astrochemically relevant radicals and radical cations characterized in the matrix isolation studies performed in our laboratory. It should be noted that the spectroscopic characterization of most of these species generated by different methods under matrix isolation conditions was reported previously due to extensive work of many groups through several decades (one may address relevant reviews [12,30,34,65]). The main purpose of Table 1 is to demonstrate the production of different radicals (potential building blocks in astrochemistry) under high-energy irradiation of the molecules detected in space in the model frozen dilute systems.

## 4. Radical–Molecule Complexes

### 4.1. Identification of Radical–Molecule Complexes and Their Possible Role in Cold Astrochemistry

The radiation-induced synthesis of COMs in icy media at cryogenic temperatures occurs under the conditions of extremely frozen molecular mobility. In this situation, the pre-existing building blocks consisting of two or more molecules should play a crucial role. In order to understand the basic mechanisms involved in the processes of cold synthetic transformations, we have introduced a general concept based on model studies of the radiation chemistry of 1:1 intermolecular complexes isolated in rigid inert media [94,95,112,117,125,134,136,137,138,139,140,141]. It should be emphasized that the interaction in such complexes is typically rather weak (the computed interaction energy usually does not exceed 2–4 kcal/mol); however, it is more than sufficient to fix the pre-determined geometry in solid cryogenic matrices, and thus, to control further radiation-induced transformations. Generally speaking, the radiation-induced processes in complexes may occur through both radical and non-radical pathways, and unequivocal judgment on their detailed mechanism is often not straightforward. Meanwhile, at least in some cases, the involvement of the open-shell intermediates (radical–molecule complexes) has been directly demonstrated [94,138,139]. The interaction in such complexes is also weak, so, in most cases, the super-hyperfine coupling with the protons of the molecular partner of the radical in the complex is small, and the corresponding structure is not revealed in the EPR spectra (some examples of a relatively strong interaction to note directly manifested in the EPR spectra was reported for fluorine-containing species [142]). The assignment of weak intermolecular and radical–molecule complexes in matrices and the identification of their structure can be performed using IR spectroscopy [136,138]. In this case, it is very important to make a comparison with ab initio calculations. While the absolute vibrational frequencies of both isolated molecules and intermolecular complexes usually cannot be calculated with sufficient accuracy for strict comparison (due to typical neglection of anharmonicity and matrix effects), an effective approach is based on the comparison of the so-called complexation-induced shifts. These shifts are determined as differences between characteristic vibrational frequency for a specific mode in isolated molecule and molecule bound in complex (generally applicable to both closed-shell and open-shell species). The experimental and computed values of the complexation-induced shifts are often in reasonable agreement, which allows one to identify the complex structure and geometry. It should be stressed that, in many cases, the complexes of different geometry exhibit not only different magnitudes but also different signs of shift for some characteristic vibrations (that is, blue or red, with respect to non-complexed molecules), which makes the assignment convincing. Additionally, the studies of isotopic substitution are helpful.

At present, the reactions occurring within radical–molecule complexes are poorly studied (with a few exceptions, such as the formation of the HOCO^•^ radical from the HO^•^⋯CO complex mentioned in Section 3.2 [93,94,95]). Meanwhile, it is really a challenging issue for different research fields, including astrochemistry, because these processes may be of key significance for the formation of some COMs in icy media, and they may occur both in the dark (even at very low temperatures) and under the action of light. A specific issue is concerned with the contribution of tunneling in such reactions, which deserves both experimental and theoretical studies.

### 4.2. The Radical–Molecule Complexes of Carbon Monoxide

Carbon monoxide (CO) is one of the widespread diatomic molecules in the ISM, which constitutes the basis for interstellar ices. It easily yields intermolecular complexes with different components under condensation of ternary mixtures M/CO/Ng. Despite the relative weakness of binding energy in most of such complexes (typically, below 2–3 kcal/mol), they can be easily identified by IR spectroscopy due to characteristic complexation-induced shifts of both fundamental CO vibration and vibrations in the second component (M). A large number of examples of such complexes is known experimentally for the closed-shell molecules, and they are rather well described by quantum-chemical computations. Meanwhile, the radical–molecule complexes of the R^•^⋯CO are still relatively poorly studied in cryogenic matrices. One example is the HO^•^⋯CO complex, a probable precursor of the hydroxycarboxyl radical and a potentially important intermediate in interstellar chemistry (see Section 3.2). This complex was characterized by characteristic shifts of both CO and HO^•^ fundamental vibrations in different noble gas matrices [93], supported by the high-level ab initio calculations [143]. As mentioned previously, it can be generated from the intermolecular precursor complex formed by very common interstellar molecules (H_2_O and CO) under the action of VUV or X-ray irradiation [93,96]:

H_2_O⋯CO → HO^•^⋯CO (41)

The decay of this complex yielding the *trans*-HOCO^•^ radical occurs at remarkably low temperatures (at ca. 20–25 K in an argon matrix and even at 4.5 K in a neon matrix [93]).

It should be noted that there is virtually no chance of detecting this complex by EPR spectroscopy because of the orbital degeneracy of the hydroxyl radical and weak interaction in the complex.

The observations of weak complexes of carbon-centered radicals with CO are very rare. One could mention the FTIR spectroscopic identification of the CF_3_^•^⋯CO produced by dissociation of a precursor closed-shell complex between fluoroform (CHF_3_) and carbon monoxide [144]. Even though the direct astrochemical relevance of this radical–molecule complex is doubtful, this interesting example shows that the precursor geometry (confirmed by the quantum-chemical calculations) is retained in the radical–molecule complex despite the intermolecular interaction being very weak. Regarding the CH_3_^•^⋯CO complex, it was not directly spectroscopically identified in matrices, so the product of photochemical dissociation of acetyl radical should rather be described as a radical–molecule pair residing in the same matrix cage (see Section 3.1.1).

### 4.3. The Radical–Molecule Complexes of Carbon Dioxide

A number of astrochemically relevant radical–molecule complexes with carbon dioxide were characterized by FTIR spectroscopy in recent years. One interesting example is a complex of ethynyl radical (C_2_H^•^⋯CO_2_), which was prepared by UV photolysis of propiolic acid in solid argon and krypton matrices [145]:

CH≡CHCOOH + hν → [C_2_H_2_⋯CO_2_] + H^•^ → C_2_H^•^⋯CO_2_ + 2H^•^ (42)

According to the comparison with the results of ab initio calculations, the radical–molecule complex adopts the most stable parallel configuration. It is worth noting that the combination of experimental and theoretical methods [145] made it possible to assign the whole set of vibronic bands of the C_2_H^•^⋯CO_2_ complex observed in the middle and near IR region (up to ~6200 cm^−1^), which appears to be the first example of a direct manifestation of the effect of weak non-covalent interactions on vibronic transitions in radical–molecule complexes.

Some other radical–molecule complexes with carbon dioxide were prepared by the annealing-induced reaction of trapped hydrogen atoms with the corresponding closed-shell intermolecular complexes. The hydrogen atoms were first generated in matrices containing appropriate (reactive) M⋯CO_2_ complexes by VUV photolysis or radiolysis and then mobilized upon annealing the samples. In particular, the complexes of carbon dioxide with C_2_H_3_^•^ (vinyl) radical [146] and *trans*-HCNH^•^ radical [138] were obtained in such ways:

C_2_H_2_⋯CO_2_ + H^•^ → C_2_H_3_^•^⋯CO_2_ (43)

HCN⋯CO_2_ + H^•^ → *trans*-HCNH^•^⋯CO_2_ (44)

In both cases, the complexes were characterized by FTIR spectroscopy, and their structure was assigned on the basis of comparison with the results of quantum-chemical calculations at the CCSD(T) level of theory.

Generally speaking, it should be noted that the complexes of simple radicals with carbon dioxide may play an important role in the astrochemical processes in the CO_2_-rich ices. However, no direct proof of their intrinsic chemical transformations under cryogenic conditions is available, probably because of rather high energy barriers for such reactions.

### 4.4. Other Radical–Molecule Complexes of Astrochemical Significance

Very limited information is available regarding other types of radical–molecule complexes, which may serve as intermediates of cold astrochemical processes. Many radical complexes with water molecules are known, and they are considered mainly in the context of atmospheric chemistry. Meanwhile, the possible role of R^•^⋯H_2_O complexes in the radiation-induced processes in the H_2_O-based cosmic ices is questionable for several reasons. First, unlike CO and CO_2_, the water molecules are highly sensitive to ionizing and VUV radiation, yielding the HO^•^ radicals, which may react with the embedded molecules. Second, the energy transfer in the water-based ices can be rather inefficient. Finally, one should bear in mind that the guest molecules in water ices may form solvates, including several H_2_O molecules instead of 1:1 localized complexes.

Regarding the radical–molecule complexes bearing synthetic potential for cold astrochemistry, one should pay attention to the complexes of radicals with unsaturated astrochemically important molecules (such as C_2_H_2_ and C_2_H_4_). Recently, we reported FTIR evidence for the stabilization of NH_2_^•^⋯C_2_H_2_ complex in a solid argon matrix produced by X-ray irradiation from the corresponding intermolecular precursor complex NH_3_⋯C_2_H_2_ [139]. The assignment was made on the basis of comparison with the results of ab initio calculations and supported by the isotopic substitution studies. It was suggested [139] that the NH_2_^•^⋯C_2_H_2_ complex may be an important intermediate in the radiation-induced transformations leading to various C,N-containing molecules.

## 5. Conclusions and Perspectives

While the simulations of chemical processes in cosmic ices attracted growing attention in the past decades, the detailed knowledge of the spectroscopic properties and chemical dynamics of the open-shell species involved in such transformations is still lacking. Beyond any doubt, such species (radicals and radical ions) are vitally important as building blocks in the nanotechnology of Nature, which actually combines “top-down” and “bottom-up” approaches for the synthesis of COMs in the space media. Obviously, various COMS (including biologically important molecules) could result from radical–radical combination. Meanwhile, other radical reactions, such as the addition of unsaturated molecules, isomerization, H abstraction, and disproportionation, may also be important under space conditions.

This review provides an outline of recent results on radicals produced under high-energy irradiation of the isolated astrochemically relevant molecules in rigid, inert matrices, which may be useful for a better understanding of the mechanisms involved in both synthesis and degradation of COMs occurring under space conditions at cryogenic temperatures. An important point is that matrix isolation helps to detect highly reactive primary radicals, which are often elusive in laboratory ice studies. Furthermore, it should be stressed that the application of matrix isolation to cold astrochemistry is not restricted to resolving spectroscopic issues, but it may really provide a bridge between computational studies (mainly neglecting the environment and using many oversimplifications) and model laboratory ice experiments typically dealing with very complex systems. A specific example of such an approach is related to the studies of the evolution of isolated intermolecular complexes, which may include the formation of radical–molecule complexes as the key intermediates. The properties and reaction pathways of the latter species are still poorly studied, and this is a challenge for both experiment and theory.

Considering the methods of characterization of the open-shell species presumably involved in astrochemical processes, vibrational (IR) spectroscopy was the primary technique used in matrix isolation studies of neutral radicals and radical–molecule complexes described in this review. On the other hand, the astrochemically important organic radical cations were mainly characterized by EPR spectroscopy. It is clear that the combination of IR and EPR spectroscopy could provide the most detailed and unequivocal information on the structure and dynamics of both neutral and charged radical species, which is important for the elucidation of the reaction mechanisms and verification of the theoretical predictions in astrochemistry and other research fields.

## Figures and Tables

**Table 1:** Radicals and radical cations generated from isolated astrochemically relevant organic molecules under irradiation with X-rays and fast electrons in solid noble gas matrices in the experiments performed at MSU and KIPC.

Radical	Source Molecule	Detection Method	Reference
CH_3_^•^	CH_3_OH	FTIR	[36]
	CH_3_CHO	FTIR, EPR	[38,39]
	CH_3_CH_2_OH	EPR	[37]
	CH_3_OCH_3_ *	EPR	[13]
	CH_3_COCH_3_ *	EPR	[38]
	CH_3_CN *	FTIR	[40]
C_2_H^•^	C_2_H_2_	FTIR, EPR	[54,56,63]
C_2_H_3_^•^	C_2_H_2_ **	FTIR, EPR	[54,56,61]
	C_2_H_4_	FTIR	[54]
	C_2_H_6_ *	FTIR	[54]
C_2_H_5_^•^	C_2_H_6_	FTIR	[54]
C_6_H_5_^•^	C_6_H_6_	FTIR, EPR	[82,83]
C_6_H_7_^•^	C_6_H_6_ **	FTIR, EPR	[82]
CH_2_OH^•^	CH_3_OH	FTIR	[36]
CH_3_CHOH^•^	CH_3_CH_2_OH	EPR, FTIR	[36,102]
HCO^•^	CH_3_OH CH_3_CH_2_OH	FTIR EPR	[36,37]
	CH_3_CHO	EPR, FTIR	[39]
HOCO^•^	HCOOH	FTIR	[96]
CH_3_CO^•^	CH_3_CHO	FTIR	[39]
HCCO^•^	CH_3_CHO *	FTIR	[39]
CN^•^	HCN	EPR, FTIR	[116]
H_2_CN^•^	HCN **	EPR, FTIR	[116]
*trans*-HCNH^•^	HCN **	FTIR	[116]
CH_2_CN^•^	CH_3_CN	FTIR	[40,122]
CH_2_NC^•^	CH_3_CN	FTIR	[40,122]
CCN^•^	CH_3_CN ***	FTIR	[122]
CNC^•^	CH_3_CN ***	FTIR	[122]
H_2_NCO^•^	NH_3_⋯CO	FTIR	[125]
NCO^•^	NH_3_⋯CO ***	FTIR	[125]
C_6_H_6_^+.^	C_6_H_6_	EPR	[41,43]
CH_3_CHO ^+.^	CH_3_CHO	EPR	[38]
CH_3_OCH_3_ ^+.^	CH_3_OCH_3_	EPR	[13]
CH_3_COCH_3_ ^+.^	CH_3_COCH_3_	EPR	[38]
*E*-HCCHCO^+.^	C_2_H_2_⋯CO	EPR, FTIR	[134]
H_2_CCCO^+.^ ****	C_2_H_2_⋯CO	EPR, FTIR	[134]

* Trace amounts. ** Appear after annealing of the irradiated samples. *** Appear at high-absorbed doses. **** Appears after subsequent photolysis of X-ray irradiated sample with visible light.

## Data Availability

No new data were created.
